# Use of Proton Pump Inhibitors and the Risk for the Development of Gastric Cancers: A Nationwide Population-Based Cohort Study Using Balanced Operational Definitions

**DOI:** 10.3390/cancers14205172

**Published:** 2022-10-21

**Authors:** Eun Jeong Gong, Chang Seok Bang, Dong-Kyu Kim, Jae Jun Lee, Gwang Ho Baik

**Affiliations:** 1 Institute of New Frontier Research, Hallym University College of Medicine, Sakju-ro 77, Chuncheon-si 24253, Gangwon-do, Korea; 2 Department of Internal Medicine, Hallym University College of Medicine, Sakju-ro 77, Chuncheon-si 24253, Gangwon-do, Korea; 3 Institute for Liver and Digestive Diseases, Hallym University, Chuncheon-si 24253, Gangwon-do, Korea; 4 Division of Big Data and Artificial Intelligence, Chuncheon Sacred Heart Hospital, Chuncheon-si 24252, Gangwon-do, Korea; 5 Department of Otorhinolaryngology-Head and Neck Surgery, Chuncheon Sacred Heart Hospital, Hallym University College of Medicine, Sakju-ro 77, Chuncheon-si 24253, Gangwon-do, Korea; 6 Department of Anesthesiology and Pain Medicine, Hallym University College of Medicine, Sakju-ro 77, Chuncheon-si 24253, Gangwon-do, Korea

**Keywords:** gastric cancer, proton pump inhibitors, cohort studies, *Helicobacter pylori*

## Abstract

**Simple Summary:**

Previous cohort studies using national claim data in Korea have shown conflicting results about the association between the use of proton pump inhibitors (PPIs) and the risk of gastric cancer. In this population-based cohort analysis using balanced operational definitions, proton pump inhibitor use was not associated with an increased risk of gastric cancer (Hazard ratio: 1.30, 95% confidence interval: 0.75–2.27). Previous cohort studies with an inappropriate operational definition for the inclusion criteria of the study subjects or index dates could be the reason of conflicting results.

**Abstract:**

Objectives: Previous cohort studies using national claim data in Korea have shown conflicting results about the association between the use of proton pump inhibitors (PPIs) and the risk of gastric cancer. This may be due to differences in the inclusion criteria or index dates of each study. This study aims to evaluate the association between PPI use and the risk of gastric cancer using balanced operational definitions. Design: A population-based cohort analysis was conducted using the Korean National Health Insurance Service database. Subjects who used PPIs or histamine-2 receptor antagonist (H_2_RA) for more than 60 days after *Helicobacter pylori* eradication were included. The study subjects were those who had never used H_2_RAs (PPI users) and controls were those who had never used PPIs (H_2_RA users). For comparison, the index dates of previous studies were adopted and analyzed. The subjects were followed until the development of gastric cancer, death, or study end. Results: A total of 10,012 subjects were included after propensity score matching. During a median follow-up of 6.56 years, PPI was not associated with an increased risk of gastric cancer (Hazard ratio: 1.30, 95% confidence interval: 0.75–2.27). This was consistent if the cumulative daily dose was adjusted (90/120/180 days), or if the index date was changed to the first day of PPI prescription or the last day of *Helicobacter pylori* eradication. There was no significant difference in mortality between both groups. Conclusion: PPI use was not associated with an increased risk of gastric cancer.

## 1. Introduction

Gastric cancer remains an important health-related burden globally, as it is the fifth most commonly occurring cancer and fourth leading cause of cancer-related death worldwide according to Global Cancer Statistics 2020 [1]. Chronic *Helicobacter pylori* (*H. pylori*) infection is considered the most powerful etiology for the development of non-cardia gastric cancer. With the decreasing trend of *H. pylori* infection worldwide, the incidence and cancer-related mortality of non-cardia gastric cancer have been steadily declining over several decades [1]. However, clinicians still encounter a substantial number of gastric cancer patients and this is presumed to be related to the increased prevalence of gastroesophageal reflux disease (with Barrett’s esophagus) and obesity, especially in the developed countries [1,2]. Another important finding is the increased incidence of gastric cancers, particularly both cardia- and non-cardia gastric cancer, in younger populations [1,3,4]. Accumulating evidence suggested that this trend was consistent in developing and developed countries [1,5,6]. Gastric cancers not related to *H. pylori* infection (*H. pylori*-negative gastric cancer) have been reported and the postulated reasons were as follows: autoimmune gastritis, other infections (such as Epstein–Barr virus), genetic factors, and gastric microbial dysbiosis, possibly related to the use of proton pump inhibitors (PPIs) or antibiotics [1,7].

Proton pump inhibitors have been widely used to manage acid-related gastrointestinal disorders. This is currently the standard treatment, not only in patients with peptic ulcers but also in patients with drug-induced gastrointestinal hemorrhage, gastroesophageal reflux disease, or functional dyspepsia. Concerns were raised about the long-term use of these agents regarding an increased risk of the development of renal diseases, bone fractures, pneumonia, dementia, small intestinal bacterial overgrowth, and gastrointestinal malignancies [8]. Although the overall quality of evidence PPI-related adverse events is relatively low, controversy persists regarding whether long-term PPI use is associated with an increased risk of developing gastric cancers [8].

In Korea, the incidence of gastric cancer is one of highest rates globally. Previous population-based cohort studies using national claim data in Korea [9,10] have shown conflicting results about the association between PPIs and the risk of gastric cancer (the study by Seo et al. [10] found that PPIs were associated with gastric cancer, while the study by Shin et al. [9] found that PPIs were not associated with gastric cancer). However, the inclusion criteria of the study subjects or index dates were not consistent, and the definition of *H. pylori* eradication and control groups differed between the two studies [9,10]. Moreover, the follow-up period was relatively short in both studies (Table 1). Considering that PPI prescription is an excellent marker of comorbidities and *H. pylori* infection is the most powerful risk factor for gastric cancer development, the conclusions drawn from previous studies need to be re-evaluated. The aim of this study is to evaluate the association between PPI use and the risk of gastric cancer using balanced operational definitions.

## 2. Methods

### 2.1. Source of Data

Data from the Korean National Health Insurance Service–National Sample Cohort (NHIS-NSC), which is a population-based cohort established by the Korean NHIS, were used for this study. Currently, the Korean NHIS maintains and stores the records of healthcare utilization and prescriptions for the entire population as a single universal government insurer [11]. The NHIS-NSC is a representative sample cohort of 1,025,340 randomly selected participants, comprising 2.2% of the total eligible Korean population in 2002, who were followed for 11 years, until 2013 [11]. The NHIS-NSC was constructed using systematically stratified random sampling with 1476 strata in the context of age, sex, and income level and the cohort was refreshed annually by adding a representative sample of newborns, sampled across 82 strata and removing subjects who were deceased or had emigrated using the 2.2% sampling rate during the follow-up period [11]. The NHIS-NSC contains information about participants’ insurance eligibility, medical treatment history, healthcare provider’s institution and general health examination for each of the 12 years [11]. This study was approved by the Institutional Review Board of the Chuncheon Sacred Heart hospital, Korea (2019-10-008).

### 2.2. Research Design

A population-based cohort analysis was conducted using the Korean NHIS-NSC data to evaluate the association between PPI use and the risk of gastric cancer using balanced operational definitions because there was substantial discrepancy in the inclusion criteria of the study subjects or index dates between two previous cohort studies analyzing the same data (Table 1) [9,10]. The balanced operational definition of PPI and histamine-2 receptor antagonist (H_2_RA) usage was those who were prescribed PPI or H_2_RA, respectively, for more than 60 days (or 90/120/180 days) after the eradication of *H. pylori.* We defined *H. pylori* eradication as therapy with a combination of amoxicillin and clarithromycin or bismuth, metronidazole, and tetracycline. The study cohort consisted of subjects who were prescribed PPI (target cohort) or H_2_RA (comparative cohort), divided according to the medication duration (60, 90, 120, and 180 days) during the index period (1 January 2003 to 31 December 2009), who were over 20 years of age at cohort entry. We established a washout period from 1 January to 31 December 2002 to remove any potential pre-existing cases of gastric cancer. Additionally, we excluded the following subjects: 1. Those in the PPI group who were prescribed any H_2_RA medication or PPI medication in the H_2_RA group during the index period; 2. Those who died as a result of any cause before 2009; 3. Those diagnosed with any malignancy before 2009; and 4. Those diagnosed with other malignancy before the diagnosis of gastric cancer. As a result, in contrast to the previous studies, the study subjects were those who had never used of H_2_RAs (PPI users) and control subjects were those who had never used PPIs (H_2_RA users). We applied 1:1 propensity score matching for the PPI and H_2_RA groups according to the medication duration (Supplementary Tables S1–S4). The operational definitions of the study endpoints were all-cause mortality or gastric cancer development. All subjects who had no event and who were alive until 31 December 2013 were censored after this time point (Supplementary Tables S5 and S6). The risk of gastric cancer was compared between PPI and H_2_RA groups using person-years at risk, which was defined as the duration between either the start of PPI or H_2_RA, and their respective endpoints (Supplementary Figure S1).

### 2.3. Statistical Analysis

We employed 1:1 propensity score matching according to sex, age, residential area, household income, and comorbidities. To adjust for comorbidities, the Charlson Comorbidity Index (CCI) was used. This is the most widely used method for comorbidity correction in big-data studies based on the International Classification of Diseases code. Specifically, in this study, we weighted the major diseases excluding gastric cancer, and all cases with a CCI score of 3 or more were defined as 3 points. The incidence rate of gastric cancer per 1000 person-years was obtained by dividing the number of subjects with gastric cancer by person-years at risk. The overall disease-free survival rate was determined using the Kaplan–Meier survival curves throughout the observation period. To determine whether there was an increased risk of gastric cancer development with PPI compared to H_2_RA, we used Cox proportional hazard regression to calculate the hazard ratio (HR) and 95% confidence interval (CI), adjusting for other predictor variables. All statistical analyses were performed using R version 3.3.1 (R Foundation for Statistical Computing, Vienna, Austria) with a significance level of 0.05.

## 3. Results

### 3.1. Study Subjects

**Figure 1:**
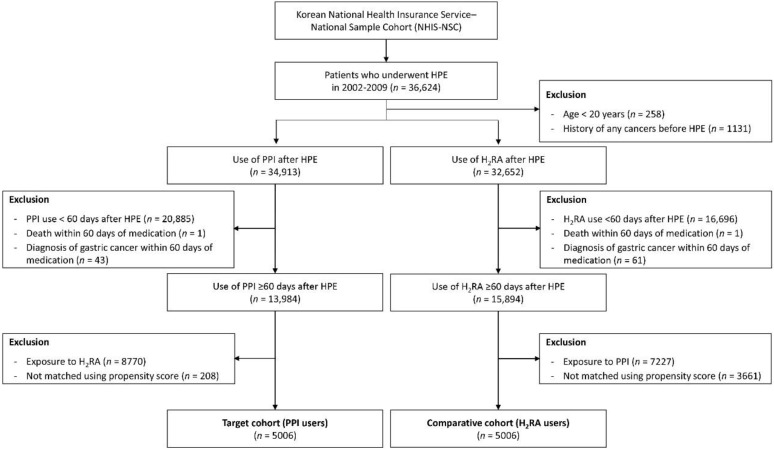
Flow diagram of study subject selection. n, number; H_2_RA, histamine-2 receptor antagonist; PPI, proton pump inhibitor; HPE, *Helicobacter pylori* eradication.

A flow chart of subject selection is shown in Figure 1. Among the 1,025,340 participants in the NHIS-NSC cohort, 36,624 subjects who underwent *H. pylori* eradication from 2002 to 2009 were included. After exclusion of participants who were under age 20 years or had a history of cancer before *H. pylori* eradication, 34,913 subjects in the target cohort (PPI users) and 32,652 in the comparative cohort (H_2_RA users) groups were enrolled. Participants who were prescribed each medication for less than 60 days, who died or were diagnosed with gastric cancer within the lag phase (60 days of starting medication) were excluded. After excluding participants who were exposed to H_2_RA in PPI group and who were exposed to PPI in H_2_RA group, propensity score matching was performed. Finally, 5006 participants were included in the PPI and H_2_RA groups.

The balance plot for five variables (sex, age, residence, household income, and CCI) before and after matching according to each cohort dataset based on the duration of medication is shown in Supplementary Figure S2. There were no significant differences in the demographic variables between PPI and H_2_RA users after propensity score matching according to the medication duration (Supplementary Figure S2 and Supplementary Tables S1–S4). The clinical characteristics of the study subjects according to each cohort dataset based on the duration of medication are described in Supplementary Tables S1–S4. Male predominance was consistently found according to the medication duration and, in the analysis by age, the proportion of subjects aged 45–64 years was higher than the other age groups. The proportion of high household income and a CCI score of zero was the highest in the overall cohort.

There were no significant differences in follow-up time in the PPI or H_2_RA groups according to the medication duration (Supplementary Figure S3 and Supplementary Tables S5 and S6).

### 3.2. Gastric Cancer Development

A total of 51 incident cases of gastric cancer were detected in the 10,012 subjects included in the cohort. The demographic characteristics of each group are described in Supplementary Tables S1–S4. During a median follow-up of 6.56 years, a cumulative daily dose of 60 days of PPI use was not associated with an increased risk of gastric cancer compared to a similar use of H_2_RA (adjusted HR: 1.30, 95% CI: 0.75–2.27) (Table 2). 

### 3.3. Sensitivity/Subgroup Analysis and Mortality

Sensitivity analysis and subgroup analysis according to the demographics or the index dates of previous studies were adopted and separately analyzed to compare the results with those of previous studies. 

**Figure 2:**
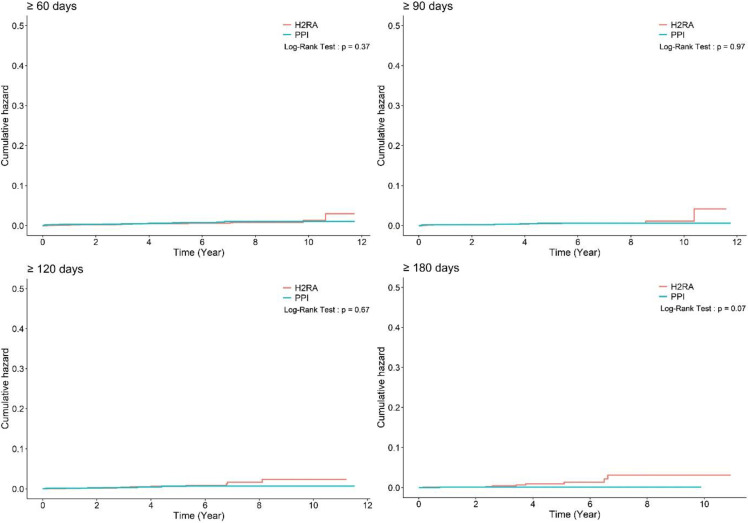
Cumulative hazard plot of gastric cancer development according to the duration of medication. H_2_RA, histamine-2 receptor antagonist; PPI, proton pump inhibitor.

First, the study subjects were analyzed based on cumulative daily medication doses of 90, 120, or 180 days and the results were consistent with the initial analysis, irrespective of the duration of PPI use (adjusted HR: 0.98, 95% CI: 0.43–2.22; adjusted HR: 0.81, 95% CI: 0.31–2.14; and adjusted HR: 0.19, 95% CI: 0.02–1.53 for more than 90, 120 and 180 days of PPI use, respectively) (Table 2 and Figure 2). 

Second, subgroup analyses according to patient sex (Supplementary Table S7), age (Supplementary Table S8), or CCI (Supplementary Table S9) consistently showed no association between increased gastric cancer risk and the use of PPI.

Third, the index dates of previous studies [9,10] were adopted and separately analyzed. The results were consistent with the initial analysis when the index date was changed to the first day of PPI prescription (adjusted HR: 1.33, 95% CI: 0.77–2.32) (Supplementary Table S10) or last day of *H. pylori* eradication (HR: 1.46, 95% CI: 0.84–2.55) (Supplementary Table S11). The results were consistent when the study subjects were analyzed based on a cumulative daily medication dose of 90, 120, or 180 days (Supplementary Tables S10 and S11).

**Figure 3:**
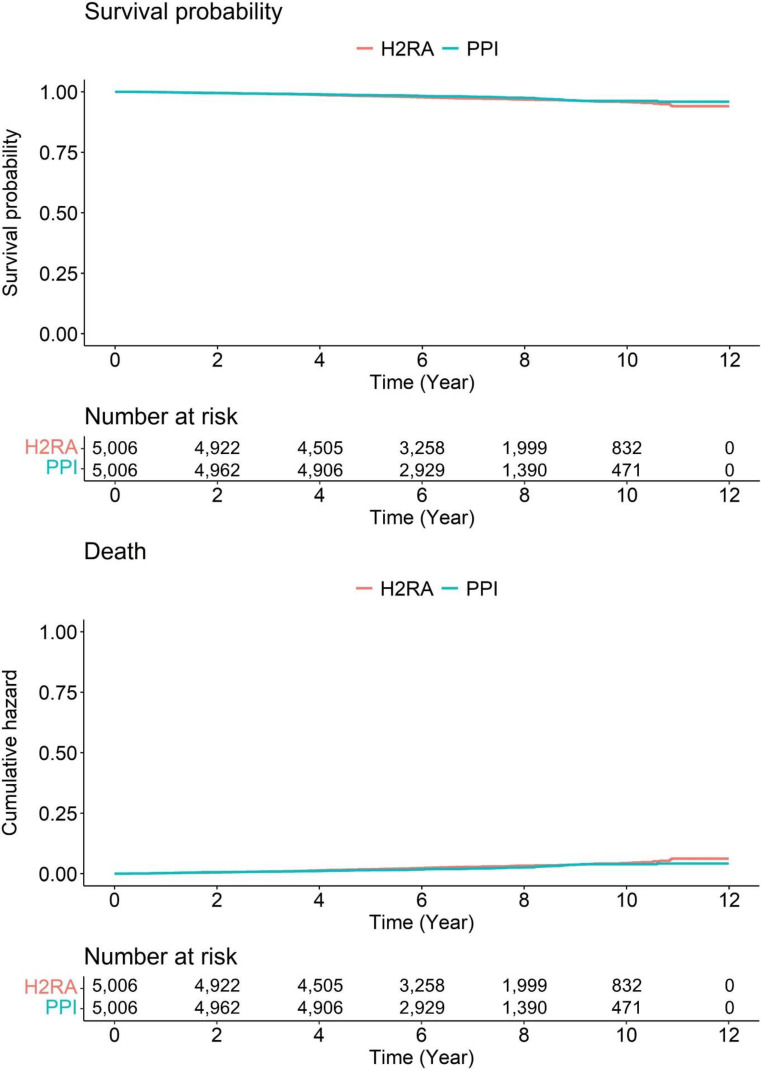
Survival curves of enrolled subjects. H_2_RA, histamine-2 receptor antagonist; PPI, proton pump inhibitor.

In the context of the mortality in the enrolled subjects, there were no data in the previous studies [9,10]. During the median follow-up of 6.56 years (7.16 year for H_2_RA group, interquartile range: 5.15–9.21 years; 6.49 years for PPI group, 5.05–8.16 years), there was no significant difference in the mortality between PPI and H_2_RA user groups (*p*-value: 0.08) (Figure 3).

## 4. Discussion

Random sampling from population and statistical analysis for characteristic features provides a good representation of an entire population. According to this nationwide population-based sample cohort study, there was no association between PPI use and the risk of gastric cancer. Our findings agree with those of a previous study that assessed data from the Korean NHIS (2002–2017) [9], but contrast with those of another recently published study that showed an association between PPI use and the risk of gastric cancer using the Korean NHIS-NSC (2002–2013) (OMOP-CDM version) and Korean NHIS-NSC (2002–2013) [10]. The difference in study findings may be due to the differences in the operational definitions of the inclusion criteria or index dates in each study, although the same basic patient data were used. Therefore, the differences in the operational definition in methodology were analyzed and a balanced operational definition was adopted in this study.

One of the key differences in the studies is that the study by Seo et al. included subjects who used PPIs for more than 30 consecutive days [10]. However, as the basic prescription duration for PPIs in patients with gastroesophageal reflux disease or functional dyspepsia is at least 2 months; therefore, this definition of PPI use is too short from a risk exposure perspective. In contrast, the study by Shin et al. [9] included subjects who used PPIs for more than 180 days (Table 1). To find the difference in both studies, we adopted a balanced operational definition of 60 days of PPI use in this study and performed a sensitivity analysis by extending the duration of use to 90, 120, or 180 days. We found no association between PPI use and the risk of gastric cancer for any of these durations (Table 2 and Figure 2).

*H. pylori* infection is the most powerful risk factor for the development of gastric cancer. While previous studies [9,10] matched and adjusted for *H. pylori* infection (and the study by Seo et al. [10] additionally analyzed subjects who had *H. pylori* eradication), PPI use duration was relatively short at more than 30 days and the mixed use of PPIs and H_2_RA was not excluded. Therefore, we only included subjects who had documented therapy for *H. pylori* eradication to exclude the most powerful confounding factor, in order to confirm the association between PPI use and gastric cancer (Table 1).

Both previous studies [9,10] adopted an active comparator over a nonuser comparison group to reduce the protopathic bias (the study by Seo et al. [10] additionally adopted control subjects who were taking any medications in addition to PPI). However, the co-prescription or alternative prescription of PPI with H_2_RA could not be totally excluded in those comparisons. The utilization patterns of acid suppressants in real-world situations could be heterogenous, that is, PPI and H_2_RA alternative consumption is possible. Therefore, this study selected subjects who had never used H_2_RA (pure PPI users) and a control group of those who never used PPIs (pure H_2_RA users) to overcome this issue (Table 1).

Based on the balanced operational definitions stated above, this study confirmed that there is no association between PPI use and gastric cancer development. The sensitivity analysis and subgroup analysis according to the demographics, study subjects, or the index dates used in the previous studies [9,10] showed results consistent with the initial analysis. The study subjects were analyzed based on cumulative daily medication doses of 90, 120, or 180 days and the results are consistent with the initial analysis, irrespective of the duration of PPI use (adjusted HR: 0.98, 95% CI: 0.43–2.22; adjusted HR: 0.81, 95% CI: 0.31–2.14; and adjusted HR: 0.19, 95% CI: 0.02–1.53 for more than 90, 120 and 180 days of PPI use, respectively) (Table 2 and Figure 2). Rather, the adjusted HR tends to decrease as the cumulative dose increases. Moreover, only our study conducted an analysis of mortality and subsequently found no significant difference between PPI and H_2_RA use. Additionally, the follow-up duration for the entire cohort was the longest in our study, with a median 6.56 years compared to those of the previous studies (median 4.2 years in the study by Shin et al. [9] and median 4.3 years in the study by Seo et al. [10]).

Although plausible hypotheses exist for the association between PPI use and gastric cancer development, such as hypergastrinemia (enterochromaffin-like cell hyperplasia) or dysbiosis (non-*H. pylori* bacterial overgrowth, especially for bacteria producing N-nitrosamine compound) induced by prolonged gastric acid suppression, or increased risk of gastric cancer with co-existing *H. pylori* infection (worsening of gastric atrophy) [12,13,14,15], there have been few case reports of a gastric cancer associated with PPI use [16]. In contrast to the association between gastric carcinoids and gastric acid suppression [17,18], real-world data are lacking in the association between PPI use and the risk of gastric cancer. Although PPIs have been used for almost 30 years worldwide, the lack of case reports describing sporadic gastric cancer induced by PPI use raises questions about whether there is a genuine risk [19]. A recently published nested case–control study in a large, community-based integrated healthcare setting also showed no association between more than two years of PPI use and increased risks of different gastrointestinal cancers, including gastric cancer [20].

Although this study has strengths as stated above, several limitations were found in this study. In contrast to the study using Korean NHIS (2002–2017) [9], which provides entire national records for healthcare utilization and prescriptions, our study used the NHIS-NSC, which was constructed by systematic stratified random sampling with proportional allocation within each stratum [11]. Therefore, a relatively small number of study subjects were enrolled. However, the stratified proportional sample (2%) of the total eligible Korean population provides large-scale, extensive, and stable data. Moreover, propensity score matching, and extensive subgroup or sensitivity analyses revealed consistent results. Another limitation is that we cannot confirm whether *H. pylori* eradication was successful as the information of confirmative diagnostic tests for *H. pylori* are not included in this database. However, because eradication success is not obtained from the claim database, this is a limitation of the Korean national claim database. This is a common limitation of research that uses all of Korea’s national claims databases. This is not a significant limitation of our study because we analyzed the reasons of different outcomes of studies targeting the Korean national claim database.

## 5. Conclusions

In conclusion, PPI use was not associated with an increased risk of gastric cancer, irrespective of the various definitions of PPI usage or index dates for use. 

## Figures and Tables

**Table 1:** Comparison of operational definitions and characteristics of previous population-based cohort studies with those of the current study.

Parameters	Current Study	Seo SI et al. [10]	Shin G-Y et al. [9]
Claim database used in each study	Korean NHIS-NSC (2002–2013)	1. Korean NHIS-NSC (2002–2013) (OMOP-CDM version): for general population 2. Korean NHIS-NSC (2002–2013): for patients with *H. pylori* eradication	Korean NHIS (2002–2017)
Study subjects	PPI use for more than 60 days (only patients with *H. pylori* eradication)	PPI use for more than 30 consecutive days (matched and adjusted for *H. pylori* infection and patients with *H. pylori* eradication were separately analyzed)	PPI use for more than 180 days (matched and adjusted for *H. pylori* infection)
Possibility of the co-prescription in the study subjects	Study subjects are composed of never user of H_2_RA (pure PPI users)	Co-prescription or alternative prescription of PPIs with short-term H_2_RA cannot be excluded	Co-prescription or alternative prescription of PPIs with short-term H_2_RA cannot be excluded
Index date	Cumulative defined daily dose of at least 60 days (The first day of PPI prescription and the last day of *H. pylori* eradication was separately analyzed)	The first day of PPI prescription (The first day of *H. pylori* eradication was separately analyzed)	Cumulative defined daily dose of at least 180 days
Control subjects	Pure H2RA users	Subject taking any drug except PPI (Non-PPI or H2RA user was separately analyzed)	H2RA user
Definition of *H. pylori* eradication group	Who have been prescribed clarithromycin-based triple therapy or bismuth-based quadruple therapy for 7–14 days	Who have been prescribed clarithromycin-based triple therapy or bismuth-based quadruple therapy for 7–14 days	Concomitant prescription a PPI, clarithromycin, or metronidazole with amoxicillin or tetracycline
Age standard	≥20 years	≥20 years	≥40 years
Exclusion criteria to avoid protopathic bias	Prescriptions of PPIs started within 6 months before gastric cancer were excluded	Prescriptions of PPIs started within 12 months before gastric cancer were excluded	Prescriptions of PPIs started within 6 months or EGD within 1 year before gastric cancer were excluded
Censoring	1. Identification of gastric cancer or end of observation period 2. All cause death (separately analyzed)	Identification of gastric cancer or end of observation period (No mortality data)	Identification of gastric cancer or end of observation period or death (from a non-gastric cancer cause or gastric surgery) (No mortality data)
Duration of follow-up period	6.56 years (IQR: 5.05–8.16 for PPI group, 5.15–9.21 for H2RA group)	Median 4.3 years (4.4 years in the PPI group and 4.2 years in the non-PPI group)	Median 4.2 years (IQR: 2.0–6.9) in the PPI group and 4.0 years (IQR: 1.85–6.81) years in the H2RA group

NHIS-NSC, National Health Insurance Service–National Sample Cohort; OMOP-CDM, Observational Medical Outcomes Partnership–Common Data Model; PPI, proton pump inhibitor; H_2_RA, histamine-2 receptor antagonist; IQR, interquartile range, *H. pylori*, *Helicobacter pylori*.

**Table 2:** Incidence per 1000 person-years and hazard ratios of gastric cancer development between histamine-2 receptor antagonist and proton pump inhibitor user group according to the duration of medication.

Study Subjects	N	Case	Incidence	Unadjusted HR (95% CI)	Adjusted HR (95% CI)	*p*-Value
(For more than) 60 days						
H_2_RA	5006	23	1.13	1.00 (ref)	1.00 (ref)	
PPI	5006	28	1.43	1.29 (0.74–2.24)	1.30 (0.75–2.27)	0.348
90 days						
H_2_RA	2919	13	1.16	1.00 (ref)	1.00 (ref)	
PPI	2919	11	1.12	0.98 (0.44–2.22)	0.98 (0.43–2.22)	0.961
120 days						
H_2_RA	1883	11	1.64	1.00 (ref)	1.00 (ref)	
PPI	1883	7	1.21	0.81 (0.31–2.12)	0.81 (0.31–2.14)	0.677
180 days						
H_2_RA	1003	8	2.34	1.00 (ref)	1.00 (ref)	
PPI	1003	1	0.37	0.18 (0.02–1.45)	0.19 (0.02–1.53)	0.119

H_2_RA, histamine-2 receptor antagonist; PPI, proton pump inhibitor; HR, hazard ratio; CI, confidence interval.

## Data Availability

All the data are accessible and available upon request from the corresponding author. All investigators have access to the final dataset.

## Supplementary Materials

The following supporting information can be downloaded at: https://www.mdpi.com/article/10.3390/cancers14205172/s1, Table S1. Characteristics of the study subjects who took medication for more than 60 days; Table S2. Characteristics of the study subjects who took medication for more than 90 days; Table S3. Characteristics of the study subjects who took medication for more than 120 days; Table S4. Characteristics of the study subjects who took medication for more than 180 days; Table S5. Description of time to event and censored data; Table S6. Description of time to event and censored data; Table S7. Hazard ratios of gastric cancer development by sex among the included subjects; Table S8. Hazard ratios of gastric cancer development by age among the included subjects; Table S9. Hazard ratios of gastric cancer development by comorbidities among the included subjects; Table S10. Incidence per 1000 person-years and hazard ratios of gastric cancer development between histamine-2 receptor antagonist and proton pump inhibitor user group according to the duration of medication (index date was the first day of the proton pump inhibitor prescription); Table S11. Incidence per 1000 person-years and hazard ratios of gastric cancer development between histamine-2 receptor antagonist and proton pump inhibitor user group according to the duration of medication (index date was the last day of Helicobacter pylori eradication); Figure S1. Diagram of study cohort construction; Figure S2. Balance plot for 5 variables before and after matching according to each cohort dataset based on the duration of medication; Figure S3. Duration of follow-up period according to each cohort dataset based on the duration of medication.
