# Supplementary material for: Use of Proton Pump Inhibitors and the Risk for the Development of Gastric Cancers: A Nationwide Population-Based Cohort Study Using Balanced Operational Definitions
Source: Cancers (Basel). 2022 Oct 21;14(20):5172. doi: 10.3390/cancers14205172 (PMC9600864; doi:10.3390/cancers14205172)
Supplement: Supplementary file 1 [file cancers-14-05172-s001.zip › cancers-1967889-supplementary.pdf]

Supplementary

Use of Proton Pump Inhibitors and the Risk for the Development of Gastric Cancers: A Nationwide Population-Based Cohort Study Using Balanced Operational Definitions

Table S1. Characteristics of the study subjects who took medication for more than 60 days.

Variables	H_2RA (n = 5,006)	PPI (n = 5,006)	*P* value
**Sex**			>0.99
Male	3344 (66.8%)	3344 (66.8%)	
Female	1662 (33.2%)	1662 (33.2%)	
**Ages (years)**			>0.99
<45	1924 (38.4%)	1924 (38.4%)	
45-64	2657 (53.1%)	2657 (53.1%)	
>64	425 (8.5%)	425 (8.5%)	
**Residence**			>0.99
Seoul	1101 (22.0%)	1101 (22.0%)	
Second area	1364 (27.2%)	1364 (27.2%)	
Third area	2541 (50.8%)	2541 (50.8%)	
**Household income**			>0.99
Low (0–30%)	929 (18.6%)	929 (18.6%)	
Middle (30–70%)	1776 (35.5%)	1776 (35.5%)	
High (70–100%)	2301 (46.0%)	2301 (46.0%)	
**CCI**			>0.99
0	3296 (65.8%)	3296 (65.8%)	
1	911 (18.2%)	911 (18.2%)	
≥2	799 (16.0%)	799 (16.0%)	

H_2RA, histamine-2 receptor antagonist, PPI, proton pump inhibitor; Seoul, the largest metropolitan area; second area, other metropolitan cities; third area, other areas; CCI, Charlson comorbidity index.

Table S2. Characteristics of the study subjects who took medication for more than 90 days.

Variables	H_2RA (n = 2,919)	PPI (n = 2,919)	*P* value
**Sex**			>0.99
Male	1964 (67.3%)	1964 (67.3%)	
Female	955 (32.7%)	955 (32.7%)	
**Ages (years)**			>0.99
<45	1002 (34.3%)	1002 (34.3%)	
45-64	1645 (56.4%)	1645 (56.4%)	
>64	272 (9.3%)	272 (9.3%)	
**Residence**			>0.99
Seoul	659 (22.6%)	659 (22.6%)	
Second area	782 (26.8%)	782 (26.8%)	
Third area	1478 (50.6%)	1478 (50.6%)	
**Household income**			>0.99
Low (0–30%)	576 (19.7%)	576 (19.7%)	
Middle (30–70%)	991 (33.9%)	991 (33.9%)	
High (70–100%)	1352 (46.3%)	1352 (46.3%)	
**CCI**			>0.99
0	1857 (63.6%)	1857 (63.6%)	
1	572 (19.6%)	572 (19.6%)	
≥2	490 (16.8%)	490 (16.8%)	

H_2RA, histamine-2 receptor antagonist; PPI, proton pump inhibitor; Seoul, the largest metropolitan area; second area, other metropolitan cities; third area, other areas; CCI, Charlson comorbidity index.

Table S3. Characteristics of the study subjects who took medication for more than 120 days.

Variables	H_2RA (n = 1,883)	PPI (n = 1,883)	*P* value
**Sex**			>0.99
Male	1288 (68.4%)	1288 (68.4%)	
Female	595 (31.6%)	595 (31.6%)	
**Ages (years)**			>0.99
<45	592 (31.4%)	592 (31.4%)	
45-64	1097 (58.3%)	1097 (58.3%)	
>64	194 (10.3%)	194 (10.3%)	
**Residence**			>0.99
Seoul	415 (22.0%)	415 (22.0%)	
Second area	508 (27.0%)	508 (27.0%)	
Third area	960 (51.0%)	960 (51.0%)	
**Household income**			>0.99
Low (0–30%)	386 (20.5%)	386 (20.5%)	
Middle (30–70%)	650 (34.5%)	650 (34.5%)	
High (70–100%)	847 (45.0%)	847 (45.0%)	
**CCI**			>0.99
0	1173 (62.3%)	1173 (62.3%)	
1	374 (19.9%)	374 (19.9%)	
≥2	336 (17.8%)	336 (17.8%)	

H_2RA, histamine-2 receptor antagonist; PPI, proton pump inhibitor; Seoul, the largest metropolitan area; second area, other metropolitan cities; third area, other areas; CCI, Charlson comorbidity index.

Table S4. Characteristics of the study subjects who took medication for more than 180 days.

Variables	H_2RA (n = 1,003)	PPI(n = 1,003)	*P* value
**Sex**			>0.99
Male	689 (68.7%)	689 (68.7%)	
Female	314 (31.3%)	314 (31.3%)	
**Ages (years)**			>0.99
<45	265 (26.4%)	265 (26.4%)	
45-64	622 (62.0%)	622 (62.0%)	
>64	116 (11.6%)	116 (11.6%)	
**Residence**			>0.99
Seoul	220 (21.9%)	220 (21.9%)	
Second area	264 (26.3%)	264 (26.3%)	
Third area	519 (51.7%)	519 (51.7%)	
**Household income**			>0.99
Low (0–30%)	212 (21.1%)	212 (21.1%)	
Middle (30–70%)	356 (35.5%)	356 (35.5%)	
High (70–100%)	435 (43.4%)	435 (43.4%)	
**CCI**			>0.99
0	616 (61.4%)	616 (61.4%)	
1	200 (19.9%)	200 (19.9%)	
≥2	187 (18.6%)	187 (18.6%)	

H_2RA, histamine-2 receptor antagonist; PPI, proton pump inhibitor; Seoul, the largest metropolitan area; second area, other metropolitan cities; third area, other areas; CCI, Charlson comorbidity index.

Table S5. Description of time to event and censored data.

For more than 60 days use	The number of event
**Event**	51
H_2RA	23
PPI	28
**Total censored (No event)**	9961
H_2RA	4983
PPI	4978
**Termination of study**	9714
H_2RA	4841
PPI	4873
**Loss to follow up / Drop-out**	247
H_2RA	142
PPI	105
**For more than 90 days use**	**The number of event**
**Event**	24
H_2RA	13
PPI	11
**Total censored (No event)**	5814
H_2RA	2906
PPI	2908
**Termination of study**	5662
H_2RA	2815
PPI	2847
**Loss to follow up / Drop-out**	152
H_2RA	91
PPI	61

H_2RA, histamine-2 receptor antagonist; PPI, proton pump inhibitor.

Table S6. Description of time to event and censored data.

For more than 120 days use	The number of event
**Event**	18
H_2RA	11
PPI	7
**Total censored (No event)**	3748
H_2RA	1872
PPI	1876
**Termination of study**	3645
H_2RA	1807
PPI	1838
**Loss to follow up / Drop-out**	103
H_2RA	65
PPI	38
**For more than 180 days use**	**The number of event**
**Event**	9
H_2RA	8
PPI	1
**Total censored (No event)**	1997
H_2RA	995
PPI	1002
**Termination of study**	1943
H_2RA	961
PPI	982
**Loss to follow up / Drop-out**	54
H_2RA	34
PPI	20

H_2RA, histamine-2 receptor antagonist; PPI, proton pump inhibitor.

Table S7. Hazard ratios of gastric cancer development by sex among the included subjects.

Sex	Male		Female	
	H_2RA	PPI	H_2RA	PPI
**(For more than) 60 days**				
Unadjusted HR (95% CI)	1.00 (ref)	1.47 (0.76-2.86)	1.00 (ref)	0.95 (0.34-2.64)
Adjusted HR (95% CI)	1.00 (ref)	1.46 (0.75-2.84)	1.00 (ref)	1.01 (0.36-2.83)
**90 days**				
Unadjusted HR (95% CI)	1.00 (ref)	0.89 (0.33-2.43)	1.00 (ref)	1.29 (0.31-5.43)
Adjusted HR (95% CI)	1.00 (ref)	0.90 (0.33-2.47)	1.00 (ref)	1.28 (0.29-5.56)
**120 days**				
Unadjusted HR (95% CI)	1.00 (ref)	0.83 (0.26-2.66)	1.00 (ref)	0.80 (0.14-4.71)
Adjusted HR (95% CI)	1.00 (ref)	0.84 (0.26-2.66)	1.00 (ref)	0.85 (0.14-5.25)
**180 days**				
Unadjusted HR (95% CI)	1.00 (ref)	0.00 (0-Inf)	1.00 (ref)	0.72 (0.06-8.49)
Adjusted HR (95% CI)	1.00 (ref)	0.00 (0-Inf)	1.00 (ref)	0.96 (0.06-15.44)

H_2RA, histamine-2 receptor antagonist; PPI, proton pump inhibitor; HR, hazard ratio; CI, confidence interval.

Table S8. Hazard ratios of gastric cancer development by age among the included subjects.

Ages	<45		45-64		>64	
	H_2RA	PPI	H_2RA	PPI	H_2RA	PPI
**(For more than) 60 days**						
Unadjusted HR (95% CI)	1.00 (ref)	1.02 (0.25-4.06)	1.00 (ref)	1.55 (0.78-3.08)	1.00 (ref)	0.84 (0.23-3.14)
Adjusted HR (95% CI)	1.00 (ref)	1.03 (0.26-4.13)	1.00 (ref)	1.56 (0.78-3.10)	1.00 (ref)	0.80 (0.21-3.00)
**90 days**						
Unadjusted HR (95% CI)	1.00 (ref)	1.02 (0.06-16.23)	1.00 (ref)	1.01 (0.39-2.61)	1.00 (ref)	1.01 (0.14-7.15)
Adjusted HR (95% CI)	1.00 (ref)	1.04 (0.07-16.67)	1.00 (ref)	1.00 (0.38-2.60)	1.00 (ref)	0.99 (0.14-7.03)
**120 days**						
Unadjusted HR (95% CI)	1.00 (ref)	1.00 (0.06-16.06)	1.00 (ref)	0.92 (0.32-2.66)	1.00 (ref)	0.00 (0-Inf)
Adjusted HR (95% CI)	1.00 (ref)	1.04 (0.06-16.59)	1.00 (ref)	0.91 (0.31-2.63)	1.00 (ref)	0.00 (0-Inf)
**180 days**						
Unadjusted HR (95% CI)	NA	NA	1.00 (ref)	0.19 (0.02-1.54)	NA	NA
Adjusted HR (95% CI)	NA	NA	1.00 (ref)	0.19 (0.02-1.53)	NA	NA

H_2RA, histamine-2 receptor antagonist; PPI, proton pump inhibitor; HR, hazard ratio; CI, confidence interval, NA; non-applicable.

Table S9. Hazard ratios of gastric cancer development by comorbidities among the included subjects.

CCI	0		1		≥2	
	H_2RA	PPI	H_2RA	PPI	H_2RA	PPI
**(For more than) 60 days**						
Unadjusted HR (95% CI)	1.00 (ref)	1.74 (0.76-3.99)	1.00 (ref)	1.03 (0.38-2.73)	1.00 (ref)	1.06 (0.32-3.53)
Adjusted HR (95% CI)	1.00 (ref)	1.75 (0.76-4.03)	1.00 (ref)	1.02 (0.38-2.72)	1.00 (ref)	1.08 (0.32-3.63)
**90 days**						
Unadjusted HR (95% CI)	1.00 (ref)	1.74 (0.48-6.29)	1.00 (ref)	0.66 (0.16-2.76)	1.00 (ref)	0.69 (0.12-3.99)
Adjusted HR (95% CI)	1.00 (ref)	1.66 (0.46-5.98)	1.00 (ref)	0.65 (0.15-2.73)	1.00 (ref)	0.66 (0.11-3.86)
**120 days**						
Unadjusted HR (95% CI)	1.00 (ref)	2.07 (0.38-11.32)	1.00 (ref)	0.29 (0.03-2.63)	1.00 (ref)	0.69 (0.13-3.70)
Adjusted HR (95% CI)	1.00 (ref)	1.99 (0.36-10.88)	1.00 (ref)	0.29 (0.03-2.61)	1.00 (ref)	0.69 (0.12-3.88)
**180 days**						
Unadjusted HR (95% CI)	1.00 (ref)	0.00 (0-Inf)	1.00 (ref)	0.47 (0.05-4.56)	1.00 (ref)	0.00 (0-Inf)
Adjusted HR (95% CI)	1.00 (ref)	0.00 (0-Inf)	1.00 (ref)	0.40 (0.04-3.92)	1.00 (ref)	0.00 (0-Inf)

CCI, Charlson Comorbidity Index; H_2RA, histamine-2 receptor antagonist; PPI, proton pump inhibitor; HR, hazard ratio; CI, confidence interval.

Table S10. Incidence per 1,000 person-years and hazard ratios of gastric cancer development between histamine-2 receptor antagonist and proton pump inhibitor user group according to the duration of medication (index date was the first day of the proton pump inhibitor prescription).

Study subjects	N	Case	Incidence	Unadjusted HR (95% CI)	Adjusted HR (95% CI)	*P value*
**(For more than) 60 days**						
H_2RA	5006	23	0.64	1.00 (ref)	1.00 (ref)	
PPI	5006	28	0.83	1.34 (0.77-2.33)	1.33 (0.77-2.32)	0.309
**90 days**						
H_2RA	2919	13	0.61	1.00 (ref)	1.00 (ref)	
PPI	2919	11	0.56	0.98 (0.44-2.20)	0.98 (0.44-2.20)	0.958
**120 days**						
H_2RA	1883	11	0.79	1.00 (ref)	1.00 (ref)	
PPI	1883	7	0.55	0.80 (0.31-2.07)	0.83 (0.32-2.17)	0.701
**180 days**						
H_2RA	1003	8	1.08	1.00 (ref)	1.00 (ref)	
PPI	1003	1	0.15	0.14 (0.02-1.09)	0.14 (0.02-1.09)	0.061

H_2RA, histamine-2 receptor antagonist; PPI, proton pump inhibitor; HR, hazard ratio; CI, confidence interval.

Table S11. Incidence per 1,000 person-years and hazard ratios of gastric cancer development between histamine-2 receptor antagonist and proton pump inhibitor user group according to the duration of medication (index date was the last day of *Helicobacter pylori* eradication).

Study subjects	N	Case	Incidence	Unadjusted HR (95% CI)	Adjusted HR (95% CI)	*P value*
**(For more than) 60 days**						
H_2RA	5006	23	0.59	1.00 (ref)	1.00 (ref)	
PPI	5006	28	0.82	1.46 (0.83-2.54)	1.46 (0.84-2.55)	0.184
**90 days**						
H_2RA	2919	13	0.56	1.00 (ref)	1.00 (ref)	
PPI	2919	11	0.55	1.08 (0.48-2.43)	1.08 (0.48-2.44)	0.851
**120 days**						
H_2RA	1883	11	0.73	1.00 (ref)	1.00 (ref)	
PPI	1883	7	0.54	0.87 (0.33-2.26)	0.90 (0.34-2.36)	0.825
**180 days**						
H_2RA	1003	8	1.01	1.00 (ref)	1.00 (ref)	
PPI	1003	1	0.14	0.15 (0.02-1.17)	0.14 (0.02-1.16)	0.069

H_2RA, histamine-2 receptor antagonist; PPI, proton pump inhibitor; HR, hazard ratio; CI, confidence interval.

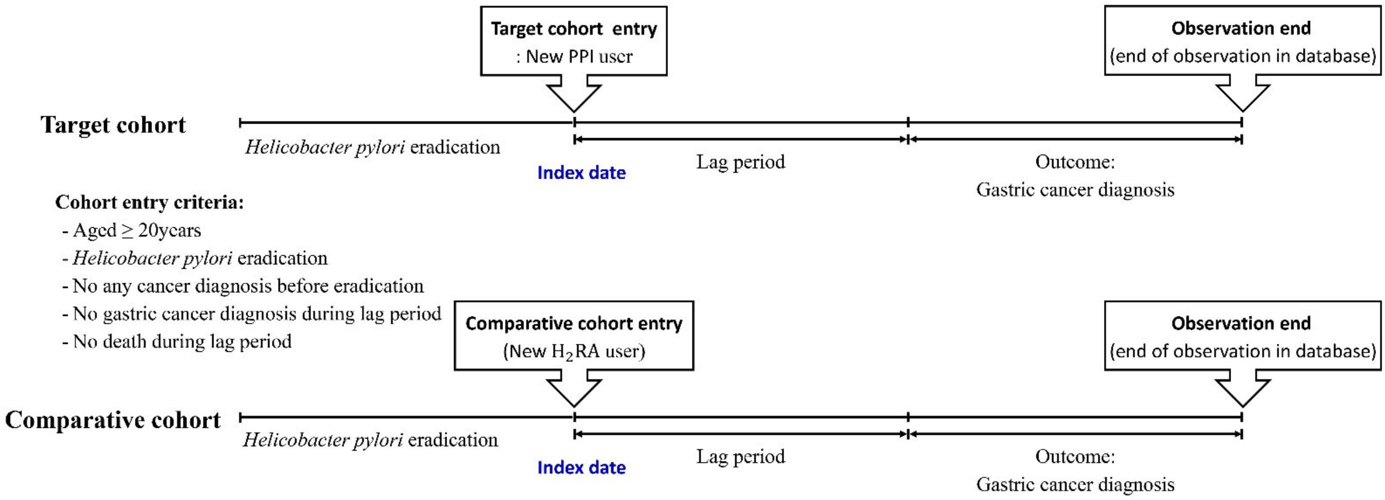

Figure S1. Diagram of study cohort construction.

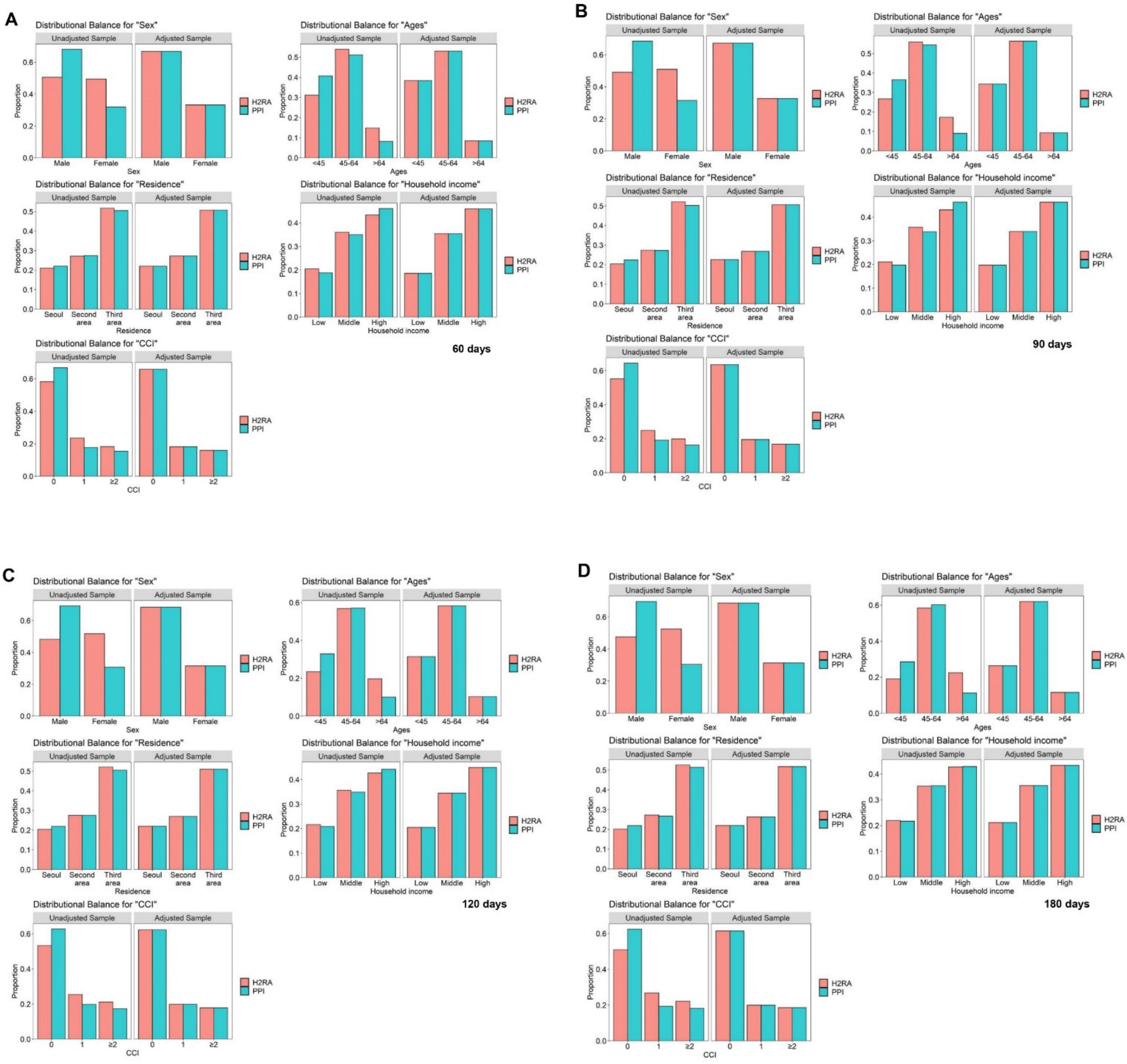

Figure S2. Balance plot for 5 variables before and after matching according to each cohort dataset based on the duration of medication. (**A**) cohort taking medications for more than cumulative daily dose of 60 days, (**B**) for more than cumulative daily dose of 90 days, (**C**) for more than cumulative daily dose of 120 days, (**D**) for more than cumulative daily dose of 180 days. H_2RA, histamine-2 receptor antagonist; PPI, proton pump inhibitor; CCI, Charlson Comorbidity Index.

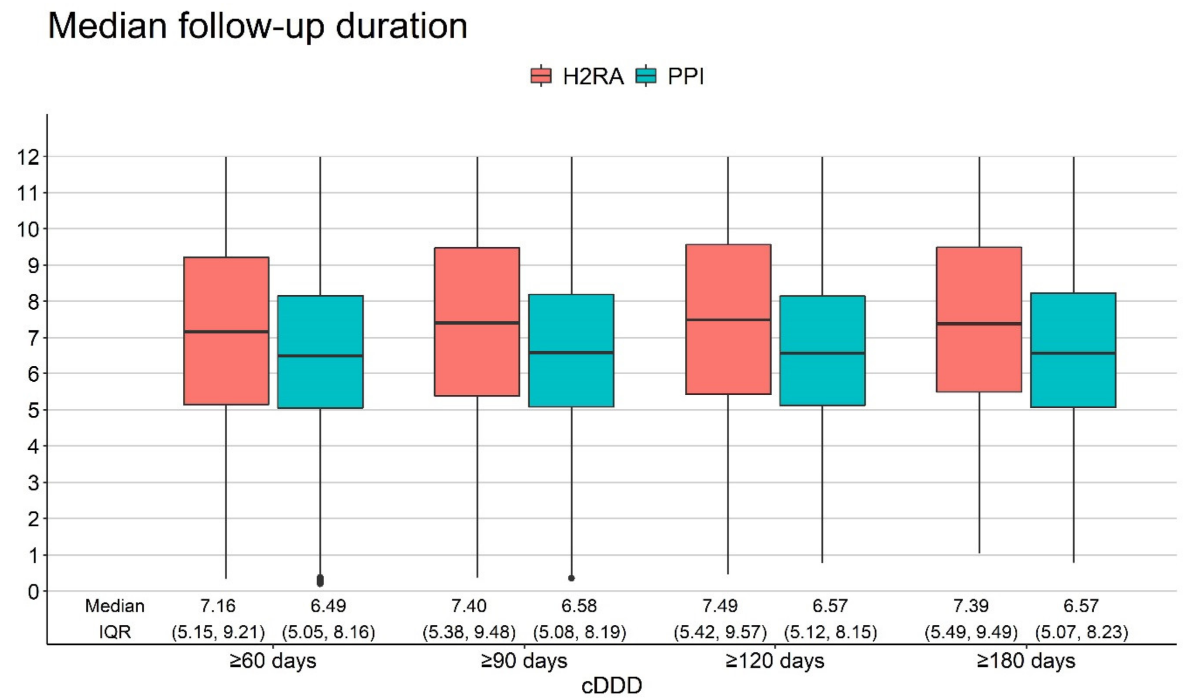

Figure S3. Duration of follow-up period according to each cohort dataset based on the duration of medication. The bottom and top edges of the box indicate the interquartile ranges. The lines in the boxes represent the median values. The lines protruding from the box indicate the range of total values. H_2RA, histamine-2 receptor antagonist; PPI, proton pump inhibitor; cDDD, cumulative drug daily dose.
